# Transcriptomic Profiling of Peripheral Blood Identifies Candidate Genes for Early Pregnancy Diagnosis in Sika Deer

**DOI:** 10.3390/ani15202960

**Published:** 2025-10-13

**Authors:** Yushi Zhang, Huimin Sun, Bingfeng Fan, Lixiang Liu, Yu Tang, Ying Zhang, Xulin Zhang, Xiaoyu Chu, Feiyu Peng, Jie Cao, Baozeng Xu

**Affiliations:** 1 Institute of Special Animal and Plant Sciences, Chinese Academy of Agricultural Sciences, Changchun 130112, China; yushi997@126.com (Y.Z.); 13994430518@163.com (H.S.); 15517046821@163.com (X.C.); pfeiyu2002@163.com (F.P.); 15229688063@126.com (J.C.); 2 State Key Laboratory for Molecular Biology of Special Economic Animals, Institute of Special Animal and Plant Sciences, Chinese Academy of Agricultural Sciences, Changchun 130112, China

**Keywords:** sika deer, pregnancy diagnosis, peripheral blood, RNA-Seq, time-series transcriptome, WGCNA

## Abstract

With estrus confined to three winter months, early pregnancy detection is essential for reproductive management in farmed sika deer. However, the development of reliable non-invasive early pregnancy detection techniques has been hindered by limited understanding of their reproductive physiology. To identify pregnancy-specific biomarkers in sika deer, we performed RNA-sequencing (RNA-Seq) on maternal peripheral blood collected on days 0, 7, 15, and 20 after artificial insemination. Using time-series clustering analysis and weighted gene co-expression network analysis (WGCNA), we identified key genes and pathways at each stage. Notably, maternal-fetal recognition-related interferon-stimulated genes (ISGs; *IFNAR1/2*, *STAT1/2*, *MX1/2*, and *RSAD2*), anti-apoptotic and immune-regulatory genes (*BCL2*, *XIAP*, and *IL10*), and cysteine metabolism genes (*CTH*, *CBS*, *GCLC*, and *GCLM*) were upregulated by day 7, suggesting their role in supporting corpus luteum development through immune regulation and redox homeostasis. By days 15–20, upregulated genes were enriched in pathways related to mitochondrial function, cell adhesion, and cell cycle regulation, indicating their involvement in embryo adhesion and syndesmochorial placentation. In conclusion, this study demonstrates that ISGs, immune-regulatory genes and cysteine metabolism genes are detectable as early as day 7 post-insemination, highlighting their promise as early pregnancy biomarkers and providing a molecular basis for non-invasive diagnostic development in sika deer.

## Simple Summary

Early pregnancy diagnosis is widely used in livestock farming for timely determining pregnancy status after insemination, which is crucial for efficient herd management and improved reproductive efficiency. Compared to traditional early pregnancy diagnostic methods such as rectal palpation and transrectal ultrasonography, non-invasive diagnostic techniques based on detecting suitable biomarkers in blood, milk, or urine enable earlier pregnancy detection with the minimal impact on reproduction. Sika deer are being increasingly farmed due to the medicinal value of their antlers, but research on early pregnancy biomarkers remains relatively limited compared to other ruminants such as cattle and sheep. In this study, we conducted transcriptome analysis of peripheral blood from four successfully pregnant sika deer on days 0, 7, 15, and 20 after artificial insemination. On day 7, we identified classic interferon-stimulated genes related to maternal recognition of pregnancy, as well as other genes involved in regulating maternal immune tolerance. On days 15 and 20, we screened genes involved in embryo implantation and early placental formation. These findings provide new references for developing early pregnancy diagnostic systems in sika deer.

## 1. Introduction

The breeding of sika deer (*Cervus nippon*) has a history of several centuries, primarily driven by the medicinal and research value of their antlers, which are known for their antitumor, anti-inflammatory, and immunomodulatory properties [1,2]. Sika deer exhibit a single-offspring birth pattern with a seasonal estrous cycle occurring from September to November [3,4]. Similarly to other ruminants, they also experience the highest rate of embryo loss during the first month of gestation [5]. Therefore, the use of assisted reproductive technologies (ART) is necessary to significantly enhance their reproductive efficiency [6]. However, challenges remain in ART, including decreased oocyte quality after in vitro maturation and reduced embryo cryotolerance, which also contribute to lower pregnancy rates [7]. This implies the importance of early pregnancy diagnosis for the timely identification of non-pregnant individuals, enabling rapid re-insemination within the limited estrous period. It also facilitates the culling of individuals with recurrent breeding failures, thereby playing a crucial role in improving reproductive efficiency at the herd level.

Conventional early pregnancy diagnostic methods, such as rectal palpation and transrectal ultrasonography, are invasive procedures that carry a risk of pregnancy loss. In ruminants, reliable diagnostic results are generally obtained at 27–45 days of gestation using these methods [8,9,10]. In sika deer, however, non-pregnant individuals typically return to estrus 18–28 days after insemination [11]. This highlights the need to develop indirect diagnostic approaches based on circulating or urinary biomarkers for earlier assessment of pregnancy status [9,12]. Given the practical difficulty of urine collection in sika deer and the ability of blood transcriptome to reflect physiological events in other tissues and organs, peripheral blood represents an ideal sample source for developing reliable diagnostic biomarkers [13]. With the rapid development of high-throughput sequencing technologies, RNA-Seq offers greater sensitivity, transcriptome coverage, and data quality for the quantitative analysis of gene expression, and its application to whole blood samples has been well validated [14,15,16].

Maternal physiological changes drive early cross-talk between the developing embryo and the uterine endometrium. In ruminants, the morula enters the uterus on days 4-6 after artificial insemination (AI) and develops into a blastocyst, which subsequently hatches and undergoes trophectoderm proliferation, leading to embryonic elongation within the first two weeks [5,17]. Embryonic elongation is also critical for sufficient secretion of interferon tau (IFNT), the signal for maternal recognition of pregnancy in ruminants. IFNT acts locally in the uterus to suppress ESR1 and OxyR expression and prevent PGF2α release, thus maintaining corpus luteum function. Additionally, it enters the peripheral circulation via the uterine vein to modulate immune cell populations and functions in concert with other immuno-regulatory molecules [18,19,20]. This coordinated activity promotes immune tolerance, uterine remodeling, and angiogenesis, thereby preventing maternal rejection of the fetal allograft and supporting placentation [21]. It also induces the expression of interferon-stimulated genes (ISGs) in maternal blood, producing transcriptomic changes that reflect early pregnancy status. Between days 15-20, the maternal system initiates embryo implantation and placentation [17,22]. The embryo first adheres to the uterine caruncles. Concurrently, uterine epithelial cells proliferate and extend numerous microvilli under estrogen stimulation. These microvilli interdigitate with those of trophoblast cells, thereby establishing tight but non-invasive junctions [17,23,24,25]. Subsequently, activated by hormones and cell cycle regulators, vascular endothelial growth factor promotes proliferation of vascular endothelial cells in the surrounding uterine tissue and induces apoptosis in adjacent stromal cells [26]. These processes ultimately lead to the formation of vascularized syncytial plaques, which constitute the structural basis of the synepitheliochorial placenta.

Importantly, mammalian pregnancy is not a state of generalized immune suppression, but rather a finely regulated balance between fetal tolerance and antimicrobial defense [27,28]. While tolerance is mediated by T-cell subsets and macrophages, antimicrobial defense relies on neutrophils and pro-inflammatory monocytes/macrophages [29,30]. During embryo development, immune regulation, and placental development, maternal energy demands increase, accompanied by enhanced mitochondrial activity [31]. Reactive oxygen species (ROS) are generated not only as byproducts of mitochondrial oxidative phosphorylation but also through metabolic reprogramming during immune responses [32,33,34]. Consequently, pregnancy is associated with activation of innate immune cells and elevated ROS production [30,35,36]. Excessive ROS accumulation, however, may induce apoptosis in luteal cells and impair placental function [37,38,39,40,41]. To counteract this oxidative stress, glutathione (GSH) serves as a major non-enzymatic antioxidant [42,43]. As precise regulation of immune balance, ROS homeostasis, and mitochondrial activity is essential for successful implantation and placentation, these processes are systematically reflected in the maternal circulation as detectable dynamic changes. Highlighting the potential of blood biomarkers as non-invasive indicators of pregnancy status.

In this study, we employed RNA-Seq to analyze the blood transcriptomes of four pregnant sika deer on days 0, 7, 15, and 20 after AI, with day 0 serving as the control. Beyond traditional pregnancy-related genes, we identified additional genes that may contribute to successful gestation by regulating cysteine metabolism, mitochondrial activity, apoptosis, and cell adhesion. These findings fill a gap in understanding the molecular mechanisms of early pregnancy in sika deer and contribute to characterizing transcriptomic changes in maternal blood, thereby identifying biomarkers that can reveal pregnancy status earlier than phenotypic traits.

## 2. Materials and Methods

### 2.1. Animals and Blood Sample Collection

Healthy and reproductively capable female sika deer (n = 10) were randomly selected for AI. Blood samples (10 mL) were collected from the jugular vein on days 0, 7, 15, and 20 after AI (designated as D0, D7, D15, and D20, respectively), placed into evacuated tubes, and stored at −80 °C. In the following spring, pregnancy status was confirmed based on successful calving. Four individuals (ID: 004, 030, 120, and 126) were identified as pregnant, and their blood samples were used for subsequent transcriptomic analysis.

### 2.2. RNA Isolation and Library Preparation

Total RNA was isolated from 1 mL whole blood using TRIzol reagent (Life Technologies, Carlsbad, CA, USA) according to the manufacturer’s instructions. Extracted RNA was stored at −80 °C until use. RNA integrity was assessed with an Agilent 2100 Bioanalyzer (Agilent Technologies, Massy, France), while RNA concentration and purity were measured using a Qubit^®^ 3.0 Fluorometer (Life Technologies, USA) and NanoDrop One spectrophotometer (Thermo Fisher Scientific, Madison, WI, USA), respectively.

Stranded mRNA libraries were prepared using the Illumina TruSeq Stranded mRNA LT Kit (Illumina, San Diego, CA, USA; Cat# RS-122-2101) following the manufacturer’s protocol. Library fragments (~370–420 bp) were size-selected using AMPure XP beads (Beckman Coulter, Brea, CA, USA). PCR amplification was then performed with Phusion High-Fidelity DNA Polymerase, Universal PCR primers, and unique index (i7) primers. The final PCR products were purified with AMPure XP beads, and library quality (fragment size distribution and adapter dimer content) was evaluated using an Agilent Bioanalyzer 2100 system.

### 2.3. Transcriptome Sequencing

RNA-sequencing was performed on the Illumina HiSeq^TM^ 3000 platform (Illumina, San Diego, CA, USA). Raw reads were processed with Trimmomatic to remove adapter-contaminated sequences and low-quality bases [44], yielding high-quality clean reads. These clean reads were then aligned to the sika deer reference genome (GWH: GWHANOY00000000, https://ngdc.cncb.ac.cn/gwh/, accessed on 13 June 2024) using HISAT2 [45]. Alignment quality was assessed using RSeQC [46], and unmapped or low-quality reads were excluded. Transcript assembly and quantification were conducted using StringTie [47]. Gene expression levels were normalized as transcripts per million (TPM) [48] based on gene length and sequencing depth, followed by log2 (TPM + 1) transformation. Principal component analysis (PCA) was performed in R (function *prcomp*), and visualizations were generated using the *ggplot2* package (V.3.3.6) [49].

### 2.4. Time-Series Clustering Analysis

To investigate global transcriptional dynamics, gene expression profiles were clustered using the fuzzy C-means (FCM) algorithm implemented in the *Mfuzz* package (V.2.52.0) in R [50]. The number of clusters was set to 6 and the fuzzification coefficient (M) was determined using the *estimate* package (V.1.0.13) in R.

### 2.5. Weighted Gene Co-Expression Network Analysis

Weighted gene co-expression network analysis (WGCNA) was performed to identify candidate biomarker genes [51]. Pairwise Pearson correlation coefficients were calculated among genes based on their FPKM values, and a topological overlap matrix (TOM) was constructed to capture both direct and indirect gene interactions. A hierarchical clustering dendrogram was then generated from TOM-based dissimilarity measures to define co-expression modules. Modules significantly associated with sampling time points were identified using a correlation threshold of |r| ≥ 0.3. For each retained module, gene significance (GS) and module membership (MM) were calculated, and their relationships with time points were visualized using clustered heatmaps.

### 2.6. Protein–Protein Interaction Network Construction

To identify hub genes with the strongest positive correlations at D7, D15, and D20, protein–protein interaction (PPI) networks were constructed using intersecting genes from the clusters and modules most correlated with each time point. Protein sequences were aligned against bovine reference sequences in the STRING database (http://www.string-db.org/)/, accessed on 6 July 2024) using BLAST (V.2.16.0), with D0 serving as the control group. PPI networks were visualized in Cytoscape (V.3.10.3), and hub genes were screened based on betweenness centrality (BC).

### 2.7. Functional Enrichment Analysis

Functional enrichment analysis was conducted for genes significantly correlated with the D7, D15, and D20 groups. Gene ontology (GO) annotation and Kyoto Encyclopedia of Genes and Genomes (KEGG) pathway analyses were performed using clusterProfiler, with GO (http://geneontology.org/, accessed on 24 July 2024) and KEGG (https://www.kegg.jp/, accessed on 26 July 2024) as reference databases. Enriched terms were considered significant if they met the criteria of *p* < 0.05 and contained at least three annotated genes.

## 3. Results

### 3.1. Overview of Transcriptome Analysis

RNA-Seq of 16 samples generated ~90 million raw reads, from which ~85 million clean reads were obtained after quality control. Over 95% of bases in the clean reads had a Phred score ≥ 30 (error rate ≤ 0.1%) with guanine–cytosine content ranging from 52.06% to 57.84%, indicating uniform base composition. On average, 79.93% of clean reads were successfully mapped to the sika deer reference genome. A total of 18,122 genes were functionally annotated through the Nr database, accounting for 84.51% of all detected genes. The high-quality sequencing data and high mapping rate confirmed its reliability for subsequent analyses.

**Figure 1:**
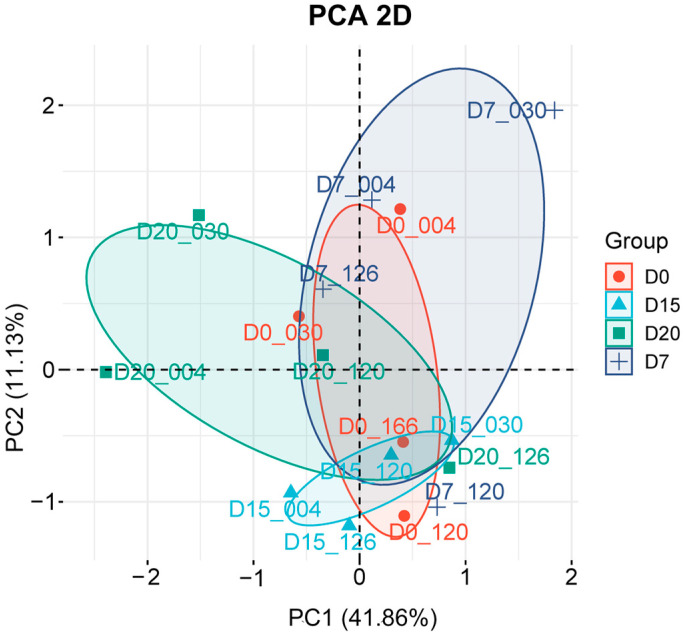
Principal component analysis (PCA) of 16 peripheral blood samples. Two-dimensional plot displays the projection of samples onto the first two principal components (PC1: 41.86%; PC2: 11.36%). Identical colors and symbols represent individual sampling times, with ellipses indicating clustering boundaries for each time point. Relative proximity between samples reflects the degree of variation, with closer distances indicating smaller differences in gene expression profiles.

PCA was performed to reduce dimensionality and visualize global variation across all 16 samples. The two-dimensional PCA plot (Figure 1) was constructed from the first two principal components (PC1: 41.86%; PC2: 11.36%). It revealed clear clustering of samples by sampling day rather than by individual variation, suggesting that pregnancy stage is the primary factor influencing transcriptional regulation. Specifically, the close clustering of D0, D7, and D15 samples reflects similar global expression profiles across these stages, However, D7 exhibits a partially distinct cluster region, reflecting unique transcriptional activity compared to both D0 and D15. In contrast, the separation of D20 samples from all earlier time points indicates pronounced transcriptomic reprogramming during later pregnancy.

### 3.2. Analysis of Transcriptome Expression Patterns

To identify genes associated with various stages of early pregnancy in sika deer, we performed time-series clustering and WGCNA on all obtained genes. By integrating the results from both methods, we systematically characterized the dynamic transcriptional profiles in maternal peripheral blood across multiple post-insemination time points.

**Figure 2:**
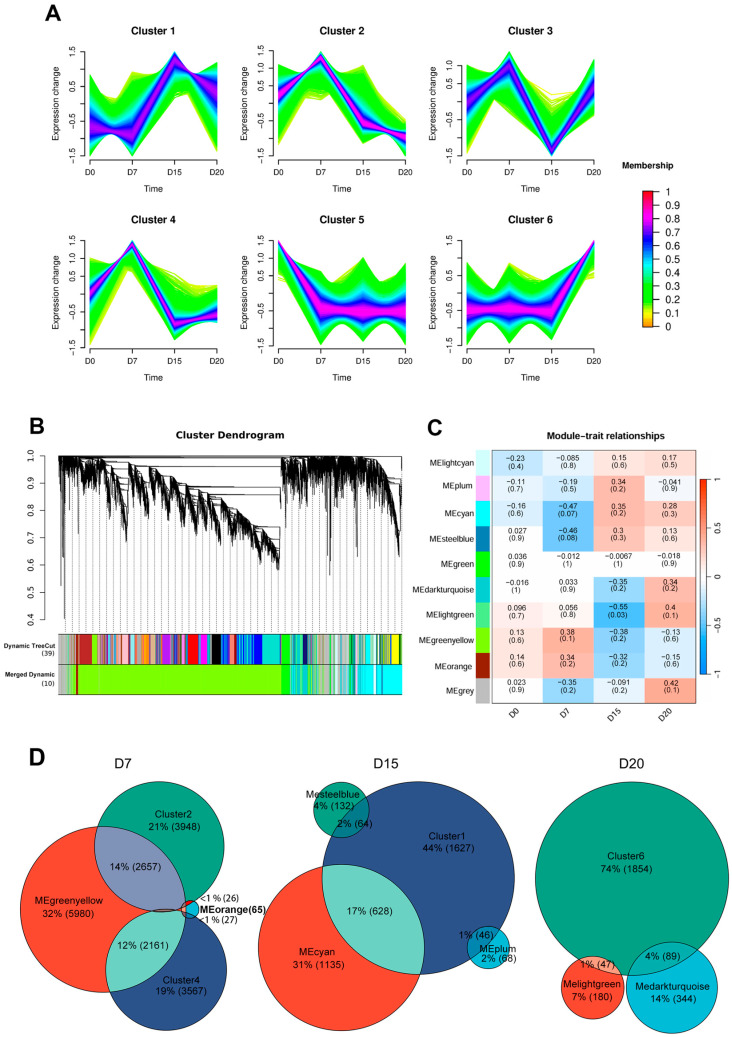
Time-series clustering analysis and weighted gene co-expression network analysis (WGCNA) of gene expression patterns. (**A**) Mfuzz-based time-series clustering identified six distinct temporal expression profiles. Genes in red show stronger conformity to the cluster center, followed by blue and green. (**B**) Hierarchical clustering dendrogram of genes based on topological overlap, with 10 co-expression modules indicated by different colors. (**C**) Heatmap showing Pearson correlations between sampling time points and module eigengenes. Module names are listed on the left, with correlation coefficients shown above each row and corresponding *p*-values in parentheses below. Row colors indicate the direction of correlation with the trait, where red represents positive and blue represents negative correlation. (**D**) Venn diagram depicting the number of overlapping intersecting genes between Mfuzz clusters and WGCNA modules most positively correlated with D7, D15, and D20 (|r| ≥ 0.3)

Time-series clustering identified six distinct gene expression patterns (Figure 2A). Clusters 2 and 4 both peaked at day 7 before declining. Cluster 1 and Cluster 6 reached maximal expression at days 15 and 20, respectively. Cluster 3 exhibited a biphasic pattern, being upregulated at day 7, downregulated at day 15, and re-upregulated at day 20. In contrast, Cluster 5 showed sustained suppression from day 7 onward compared to day 0.

WGCNA identified ten co-expression modules (Figure 2B), with the gray module (containing unassigned genes) excluded from further analysis. To assess module-time point correlations, we constructed a heatmap based on module eigengene expression across sampling days (Figure 2C). Modules most positively correlated (|r| ≥ 0.3) with specific stages included MEgreenyellow and MEorange at day 7; MEcyan and MEplum at day 15; along with MElightgreen and MEdarkturquoise at day 20.

To further investigate the dynamic regulatory network during early pregnancy, we integrated time-series clustering and WGCNA results by intersecting gene clusters and modules that showed positive correlation at each time point. This integration identified 4871, 738, and 136 intersected genes on days 7, 15, and 20, respectively (Figure 2D). These genes were subsequently analyzed to characterize stage-specific transcriptomic signatures throughout early gestation.

### 3.3. Functional Analysis of Stage-Specific Genes During Early Pregnancy

To identify hub genes specific to each gestational stage, PPI networks were constructed using STRING based on intersecting genes at D7, D15, and D20, compared with D0. Hub genes were defined as the top 50 nodes with the highest connectivity suggesting their central regulatory roles. Functional enrichment analysis of these intersecting genes was performed with the GO and KEGG databases to elucidate key biological processes and molecular mechanisms involved.

**Figure 3:**
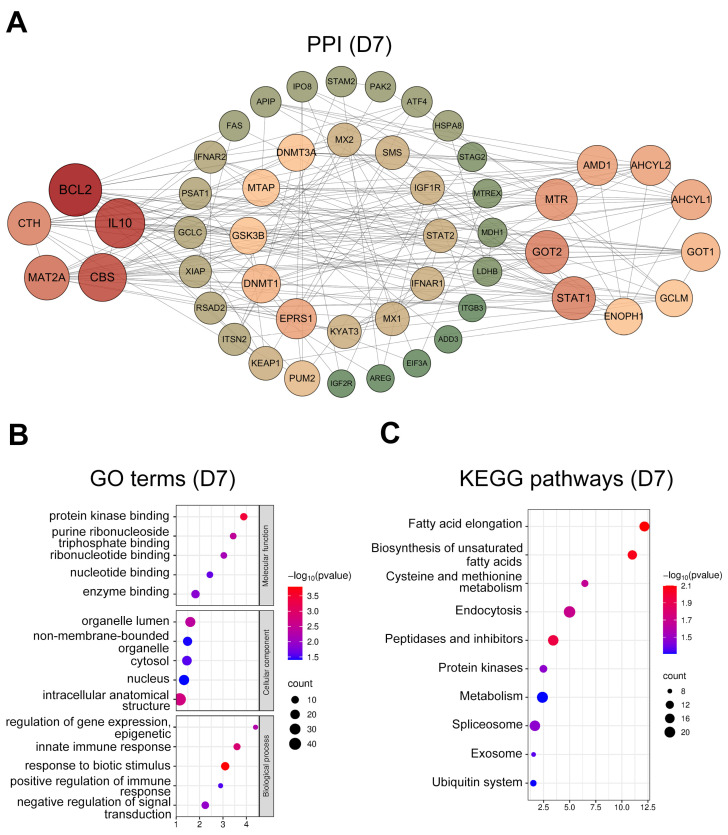
Protein-protein interaction (PPI) network and functional enrichment analysis of intersected genes at day 7. (**A**) PPI networks of the top 50 hub genes (ranked by connectivity). Node size and color intensity represent gene connectivity; (**B**) Functional enrichment analysis of stage-specific genes. The top five overrepresented Gene ontology (GO) terms in Molecular Function (MF), Cellular Component (CC), and Biological Process (BP); (**C**) The top ten significantly enriched Kyoto encyclopedia of genes and genomes (KEGG) pathways for the intersected genes.

At D7, hub genes (Figure 3A) were mainly ISGs (*IFNAR1/2*, *MX1/2*, *STAT1/2*, and *RSAD2*), immuno-regulatory and anti-apoptotic genes (*IL10*, *BCL2*, *XIAP*, and *KYAT3*), and genes involved in cysteine synthesis and metabolism (*CBS*, *CTH*, *GCLC*, *GCLM*, *MAT2A*, and *MTR*). GO enrichment (Figure 3B) of biological processes revealed significant involvement in response to biotic stimulus (GO:0009607), innate immune response (GO:0045087), and positive regulation of immune processes (GO:0050778). KEGG analysis (Figure 3C) indicated enrichment in immune-related endocytosis (ko04144), the ubiquitin system (ko04121), and cysteine and methionine metabolism (ko00270).

**Figure 4:**
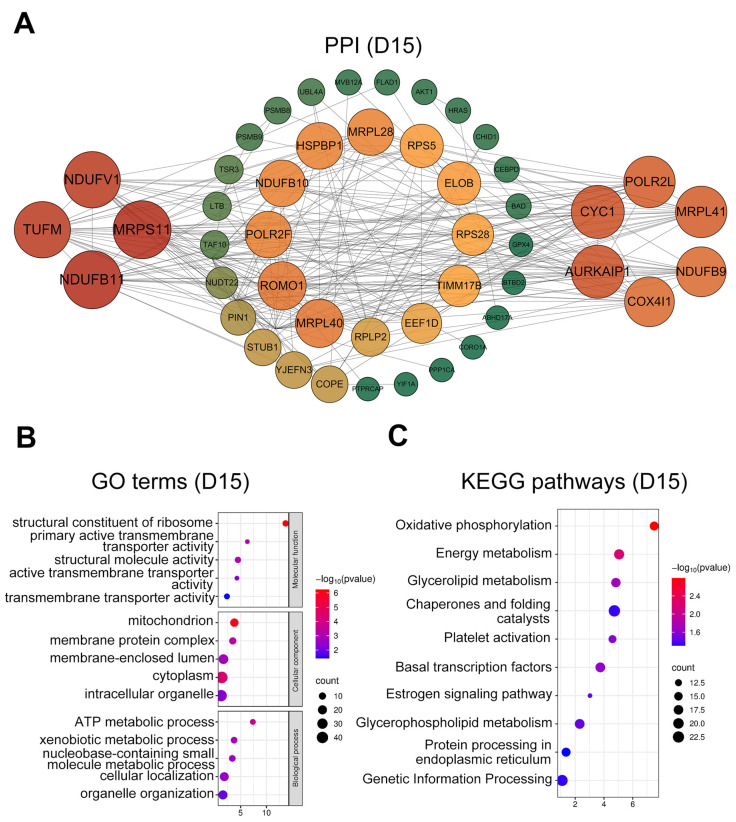
PPI network and functional enrichment analysis of intersected genes at day 15. (**A**) PPI networks of the top 50 hub genes (ranked by connectivity). Node size and color intensity represent gene connectivity; (**B**) Functional enrichment analysis of stage-specific genes. The top five significantly enriched GO terms GO terms in MF, CC, and BP; (**C**) The top ten significantly enriched KEGG pathways for the intersected genes.

At D15, hub genes (Figure 4A) were enriched for regulators of mitochondrial function and ATP production (*COX4I1*, *NDUF* family members, and *MRP* family members), together with proliferation- and differentiation-related genes (*HRAS*, *AKT1*). GO terms (Figure 4B) were predominantly enriched in intracellular organelles (GO:0043229), mitochondria (GO:0005739), cellular localization (GO:0051641), and ATP metabolic processes (GO:0046034). KEGG pathways (Figure 4C) were strongly enriched for oxidative phosphorylation (ko00190), energy metabolism (ko01100), platelet activation (ko04611), and estrogen signaling (ko04915).

**Figure 5:**
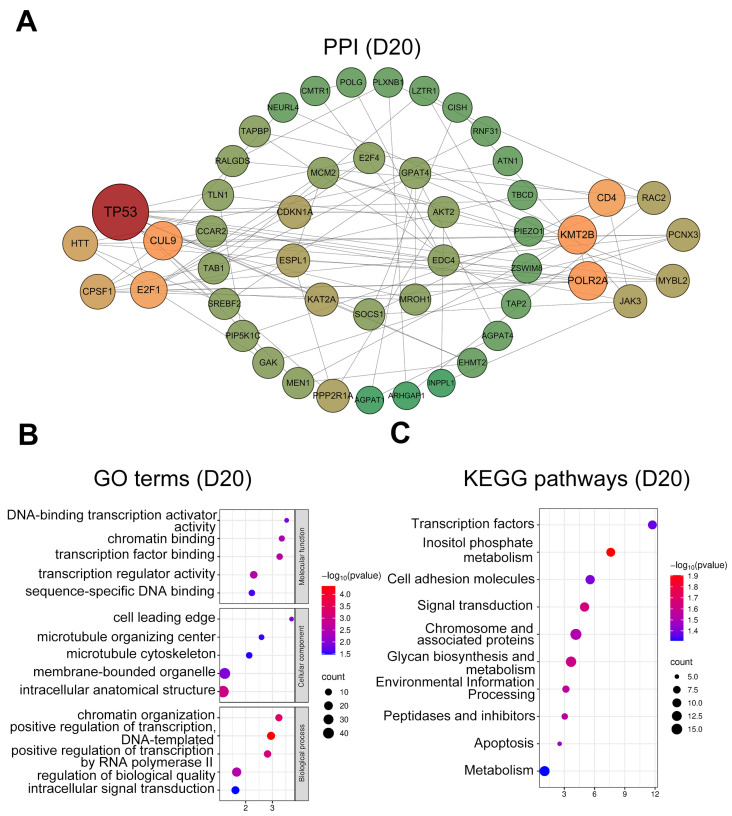
PPI network and functional enrichment analysis of intersected genes at day 20. (**A**) PPI networks of the top 50 hub genes (ranked by connectivity). Node size and color intensity represent gene connectivity; (**B**) Functional enrichment analysis of stage-specific genes. The top five significantly enriched GO terms in MF, CC, and BP; (**C**) The top ten significantly enriched KEGG pathways for the intersected genes.

At D20, hub genes (Figure 5A) were mainly related to cell adhesion (*TLN1*, *RAC2*, and *PLXNB1*) and cell cycle regulation (*CDKN1A*, *E2F1*, *MYBL2*, and *ESPL1*). GO analysis (Figure 5B) highlighted enrichment in the microtubule cytoskeleton (GO:0015630), cell leading edge (GO:0031252), transcription regulator activity (GO:0140110), and transcription factor binding (GO:0008134). KEGG pathway (Figure 5C) analysis indicated significant enrichment in signal transduction (ko04010), apoptosis (ko04210), and cell adhesion molecules (ko04514).

## 4. Discussion

Pregnancy is a dynamic process during which early gestational signals induce phenotypic and transcriptional changes in maternal peripheral blood [52]. Accordingly, blood transcriptome profiling provides a valuable approach to capture gene expression alterations during the onset of gestation. To our knowledge, this is the first attempt to use RNA-Seq to investigate transcriptomic changes during early pregnancy in sika deer. In this study, we analyzed gene expression dynamics in sika deer peripheral blood at multiple time points following AI. By integrating Mfuzz time-series clustering with WGCNA, we characterized stage-specific transcriptional patterns and interpreted gene functions through enriched biological pathways. This analysis identified candidate genes involved in maternal recognition of pregnancy, embryo implantation, and placentation, thereby revealing key regulatory mechanisms and potential biomarkers for developing early pregnancy diagnostic strategies in sika deer.

On day 7 after AI, transcriptome analysis revealed a marked upregulation of ISGs, including *IFNAR1/2*, *STAT1/2*, *MX1*/2, and *RSAD2*. Previous studies have established the significant upregulation of ISGs in the peripheral blood of ruminants; however, their detection has typically been reported at least two weeks post-AI (cattle: days 18–20, sheep: days 14–15) [53,54,55,56]. In contrast, our findings demonstrate that ISG expression is detectable in sika deer as early as day 7, indicating a more rapid maternal response. IFNT binds to type 1 interferon receptors (*IFNR1/2*) and activates the Janus kinase–signal transducer and activator of transcription (JAK/STAT) pathway [57]. JAKs initiate tyrosine phosphorylation of the receptors and recruit corresponding STATs; the phosphorylated STATs then dimerize and enter the nucleus to regulate specific gene transcription [58], such as *MX1*, *MX2*, and *RSAD2* [15,59]. These results suggest that sika deer embryos secrete sufficient IFNT within the first week after AI to trigger maternal-fetal recognition, support corpus luteum development, and produce systemic transcriptional changes detectable in peripheral blood.

In addition to ISGs, several anti-apoptotic and immune-regulatory genes (*BCL2*, *XIAP*, and *IL10*) detected in the D7 group were also identified as hub genes. GO enrichment indicated their involvement in innate immune responses and regulation of immune activity. Numerous studies demonstrate that *BCL2* and *XIAP*, as anti-apoptotic molecules, help sustain trophoblast proliferation within the immunosuppressive microenvironment [21,60,61,62,63], maintain the functional persistence of corpus luteum [64], and promote anti-inflammatory cytokine production (e.g., *IL10*, *IL4*) in macrophages [21,63]. *IL10* suppresses antigen presentation and T-cell activation, thereby limiting excessive inflammation. Elevated *IL10* levels in maternal circulation have been widely associated with successful pregnancy [65,66]. Interestingly, the cysteine and methionine metabolism pathway was also significantly enriched on day 7, with *CBS*, *CTH*, *GCLC*, and *GCLM* identified as hub genes. The enzymes encoded by these genes enhance GSH synthesis [67,68], thereby strengthening maternal antioxidant defenses. Furthermore, *CBS* and *CTH* generate hydrogen sulfide (H_2_S), a signaling molecule that promotes vasodilation, angiogenesis, and immune regulation during early pregnancy [69,70]. Previous studies in ruminants and humans suggest that estrogen-responsive CBS/H_2_S signaling contributes to uterine vasodilation, Th1/Th2 balance at the maternal–fetal interface, and modulation of trophoblast function [71,72,73,74]. Collectively, the coordinated upregulation of immune-regulatory and cysteine metabolism-related genes a D7 may help reinforce maternal tolerance and maintain ROS homeostasis, thereby supporting early pregnancy in sika deer.

In the D15 group, hub genes in the PPI network were mainly associated with mitochondrial metabolism and ATP production, including *COX4I1*, *NDUF* family genes, and *MRP* family genes. Both GO and KEGG analyses consistently revealed significant enrichment of terms and pathways related to mitochondrial activity, while KEGG also indicated enrichment in the estrogen signaling and platelet activation. At this stage, estrogen stimulates endometrial cell proliferation, thereby increasing myometrial thickness, and promotes uterine angiogenesis through the regulation of angiogenic factors [75,76]. Platelet activation, which releases multiple growth factors, further contributes to endometrial thickening and uterine receptivity [77,78]. As the primary energy source of cells, mitochondria are not only regulated by estrogen but also essential for steroid hormone synthesis in granulosa cells, thus supporting embryonic development and placentation [79,80,81]. Conversely, mitochondrial dysfunction may lead to embryonic arrest or abnormal placental vasculogenesis, ultimately resulting in implantation failure [82,83,84,85]. Together, these findings suggest that around the second week after AI in sika deer, estrogen-driven uterine remodeling, supported by mitochondrial energy supply, prepares the uterine environment for embryo adhesion and implantation.

In the D20 group, GO analysis revealed significant enrichment of terms related to biological adhesion in cellular components, including the microtubule cytoskeleton, microtubule organizing center, and cell leading edge. KEGG pathways were significantly enriched in apoptosis and cell adhesion molecules. These findings are consistent with previously reported transcriptomic data from buffalo uterine caruncles at day 45 of gestation [86] and proteomic profiles from cow urine during days 16-25 of pregnancy [87]. Such consistency likely reflects the requirement of interdigitated attachment between embryonic microvilli and uterine caruncles for placentome formation in syndesmochorial placentation. Overall, our results emphasize the central role of cell adhesion and cell cycle regulation in the early placental development of sika deer.

Despite these encouraging findings, several limitations should be acknowledged. First, the lack of non-pregnant controls makes it difficult to completely exclude time-dependent physiological changes unrelated to pregnancy. Second, the sample size was limited to four pregnant sika deer, which reduces statistical power and increases the risk that individual variation may influence the results. However, given the practical challenges of working with this species, these exploratory data still provide valuable insights. We also employed both time-series clustering analysis and WGCNA to identify key genes, which partially mitigated the risk of sample bias. Third, we cannot exclude the possibility of early embryonic loss in females that did not calve. Early embryo mortality is a known reproductive challenge in sika deer and other ruminants; however, due to insufficient follow-up sampling, we were unable to further investigate this possibility in the current study. In addition, the early upregulation of ISGs on day 7 represents a notable finding compared to reports in other ruminants. However, alternative explanations cannot be excluded, such as the high sensitivity of RNA-Seq or the influence of the limited sample size. Finally, the accuracy and feasibility of applying the candidate genes identified in this study for early pregnancy diagnostic remain to be validated.. Future studies with larger sample sizes and complementary validation approaches will be necessary to confirm and extend our findings.

## 5. Conclusions

Our findings demonstrate that ISGs, along with cysteine metabolism-related genes and genes involved in regulating maternal immune tolerance, can be detected as early as day 7 after AI in sika deer. Genes associated with mitochondrial activity, ATP production, apoptosis, and cell adhesion were primarily upregulated at days 15 and 20 post-AI, suggesting their potential roles in embryo adhesion and placentation, although the underlying mechanisms require further clarification. Overall, our findings not only validate the upregulation of known ruminant early-pregnancy biomarkers (ISGs) in sika deer, but also extend current knowledge by showing that this upregulation occurs earlier compared to cattle or sheep. Additionally, this study uncovers important roles of cysteine metabolism and mitochondrial activity in regulating early gestation. These insights provide a theoretical foundation for developing novel non-invasive diagnostic methods for early pregnancy detection in sika deer.

## Data Availability

The dataset generated and/or analyzed during the current study is available from the corresponding author on reasonable request.

## Abbreviations

The following abbreviations are used in this manuscript:

RNA-Seq	RNA-sequencing
AI	Artificial insemination
WGCNA	Weighted gene co-expression network analysis
ART	Assisted reproductive technologies
IFNT	Interferon tau
ISGs	Interferon-stimulated genes
ROS	Reactive oxygen species
GSH	Glutathione
PCA	Principal component analysis
GO	Gene Ontology
KEGG	Kyoto Encyclopedia of Genes and Genomes
GC	Guanine-cytosine
BP	Biological Process
MF	Molecular Function
CC	Cellular Component
